# An Unequal Pandemic: Vulnerability and COVID-19

**DOI:** 10.1177/00027642211003141

**Published:** 2021-11

**Authors:** Laura Robinson, Jeremy Schulz, Massimo Ragnedda, Heloisa Pait, K. Hazel Kwon, Aneka Khilnani

**Affiliations:** 1 Santa Clara University, Santa Clara, CA, USA; 2 University of California Berkeley, Berkeley, CA, USA; 3 Northumbria University, Newcastle upon Tyne, UK; 4 São Paulo State University, *Julio de Mesquita Filho*, São Paulo, Brazil; 5 Arizona State University, Phoenix, AZ, USA; 6 The George Washington University, Washington, DC, USA

**Keywords:** pandemic, COVID-19, vulnerability, inequality, crisis

## Abstract

This collection sheds light on the cascading crises engendered by COVID-19 on many aspects of society from the economic to the digital. This issue of the *American Behavioral Scientist* brings together scholarship examining the various ways in which many vulnerable populations are bearing a disproportionate share of the costs of COVID-19. As the articles bring to light, the unequal effects of the pandemic are reverberating along preexisting fault lines and creating new ones. In the economic realm, the rental market emerges during the pandemic as an economic arena of heightened socio-spatial and racial/ethnic disparities. Financial markets are another domain where market mechanisms mask the exploitative relationships between the economically vulnerable and powerful actors. Turning to gender inequalities, across national contexts, women represent an increasingly vulnerable segment of the labor market as the pandemic piles on new burdens of remote schooling and caregiving despite a variety of policy initiatives. Moving from the economic to the digital domain, we see how people with disabilities employ social media to mitigate increased vulnerability stemming from COVID-19. Finally, the key effects of digital vulnerability are heightened because the digitally disadvantaged experience not only informational inequalities but also aggravated bodily manifestations of stress or anxiety related to the pandemic. Each article contributes to our understanding of the larger mosaic of inequality that is being exacerbated by the pandemic. By drawing connections between these different aspects of the social world and the effects of COVID-19, this issue of American Behavioral Scientist advances our understanding of the far-reaching ramifications of the pandemic on vulnerable members of society.

## Introducing an Unequal Pandemic: Vulnerability and COVID-19

As of early 2021, the world has far surpassed the hundred million mark of COVID-19 cases and is approaching three million deaths globally. In addition to the cost in human lives and diminished health, there are also vast societal implications that we are only just beginning to understand. What we do know is that we have not all experienced the same pandemic. We explore these diverse phenomena across several issues of *ABS* devoted to revealing the social impacts of COVID-19. This inaugural issue opens with “Cascading Crises: Society in the Age of COVID-19” that provides a panorama of the broad array of social science scholarship examining the COVID-19 crisis from across disciplines, themes, and geographic locations. Subsequent issues devoted to the pandemic illuminate organizational and institutional responses, communication and information, politics, health and well-being, work and unemployment, and digital inequalities. Together, the whole of this work is far greater than the sum of its parts and is only possible thanks to the tremendous contributions made by dozens of international scholars and editors contributing to this project from many corners of the globe. We thank them for their collaboration and the *American Behavioral Scientist* and Laura Lawrie for allowing us to begin this journey towards what we hope will be a better post-pandemic world.

This initial issue opens with three articles exploring economic vulnerability and the fallout generated by COVID-19. The first article is “The COVID-19 Pandemic and the Rental Market: Evidence from Craigslist” by John Kuk, Ariela Schachter, Jacob William Faber, and Max Besbris. The authors examine a unique data set of rental listings culled from Craigslist matched with local public health metrics in the 49 largest metropolitan areas in the U.S. Their findings show that, although median and mean rents decline following the local spread of COVID-19 from mid-March to early June of 2020, “this trend is driven by dropping rents for listings in Black, Latino, and diverse neighborhoods. Listings in majority White neighborhoods experience rent increases during this time.” In making these linkages, their article reveals the importance of understanding the interplay of markets, race/ethnicity, and socio-spatial inequality. In a time of stay-at-home and lockdown orders, the significance of these findings cannot be understated in terms of the ethno-racial and socio-economic cleavages in all too many metropolitan areas of the United States.

Next, we turn from rental markets to financial markets in the article “Profiting on Crisis: How Predatory Financial Investors Have Worsened Inequality in the Coronavirus Crisis” by Megan Tobias Neely and Donna Carmichael. Their much-needed work reveals how U.S. shadow banks (including private equity, venture capital, and hedge fund firms) are acting as financial predators that worsen the hardship and inequality generated by COVID-19. In their words, “While investors have provided crucial funding for companies delivering healthcare innovations, protective equipment, home delivery, and remote work, shadow banks have invested in ways that also exploit the vulnerabilities and inequalities laid bare by the crisis.” Not only do the authors “identify how these investors helped to hollow out the healthcare industry and disenfranchise the low-wage service sector,” but they also show how their actions put frontline workers at risk and allow these predatory actors to “profit on the misfortunes of frontline workers, vulnerable populations, and distressed industries.” As Neely and Carmichael reveal, even as we are still battling the virus, shadow banks already are positioning themselves to profit from the continued crisis fallout heedless of the suffering of the economically vulnerable who have already “suffered the brunt of the economic and health fallouts from the pandemic.”

Subsequently, Nino Bariola and Caitlyn Collins analyze policy responses attempting to mitigate the widespread impact of the COVID-19 pandemic on the workforce in “The Gendered Politics of Pandemic Relief: Labor and Family Policies in Denmark, Germany, and the United States During COVID-19.” The article probes the plight of working mothers during the pandemic during a time in which many women are expected to shoulder the augmented burdens of homeschooling, caregiving, and challenging labor inequalities. The authors compare the public programs prioritizing childcare provisioning in Denmark and delaying the reopening of childcare facilities in Germany to the largely private sector efforts in the U.S. Their findings reveal that these contrasting policy initiatives are undergirded by fundamentally different “cultural infrastructures.” In their words: “In Denmark, a social democratic welfare state, robust federal salary guarantee programs supplemented an already strong social safety net . . . Germany, a corporatist regime, substantially expanded existing programs and provided generous subsidies . . . the U.S.’s liberal regime, private organizations—particularly in privileged economic sectors—are the ones primarily offering supports to working parents.” As they show, inadequate policy responses to the pandemic continue to have “disastrous consequences for women, especially the most vulnerable.”

From economic vulnerabilities, we move to two articles considering how different vulnerable populations fare in the digital realm. These two articles show the increased importance of digital resources and skills, as well as the impact of digital scarcities, during the pandemic. The first of the two articles is “Piercing the Pandemic Social Bubble: Disability and Social Media Use About COVID-19” by Kerry Dobransky and Eszter Hargittai. Based on an original and nationally representative data set of adult Americans, the authors examine how PWD (People with Disabilities) employ social media to exchange information about the crisis during the first wave of the pandemic in the U.S. Significantly, Dobransky and Hargittai point out that, although PWD are disproportionately vulnerable, “PWD were more engaged with information about COVID-19 than those without disabilities, even when controlling for sociodemographics and internet experiences and skills. These differences are especially pronounced concerning more active engagement such as sharing information, interacting, and supporting others on social media.” They close their article by considering the benefits of universal design for all individuals, during and beyond the pandemic, and point out that the general population stands to benefit from the “tools long fought for and used by PWD.”

Finally, the issue concludes with “The COVID Connection: Pandemic Anxiety, COVID-19 Comprehension, and Digital Confidence” by Laura Robinson, Jeremy Schulz, Øyvind Wiborg, and Elisha Johnston. Based on nationally representative data on American adults from the Pew Foundation, the authors map out how digital inequality can be implicated in both informational and somatic vulnerabilities. They find that digital confidence is a determinant of “comprehension of information about COVID-19 and anxiety related to the virus manifested by physical symptoms.” More specifically, the findings establish a linkage between digital inequality and bodily stress/anxiety related to COVID-19 (sweating, trouble breathing, nausea, or a pounding heart) experienced by respondents “when merely thinking about their experiences with the COVID-19 outbreak.”

Across these articles, it is increasingly clear that the COVID-19 crisis is truly an unequal pandemic. Society’s most vulnerable populations are disproportionately suffering in both the economic and digital realms. By drawing connections between these different aspects of inequality across the social world and the effects of COVID-19, this issue advances our understanding of the secondary and tertiary effects of the pandemic. A year into the pandemic, we are only beginning to understand how COVID-19 intersects with a multitude of preexisting and emergent vulnerabilities. As we have seen in this issue, the suffering has not been equal. Future work must uncover the manifold long-term effects of the pandemic that compound the tragic death, illness, economic devastation, and social isolation that have become all too familiar for society’s most vulnerable members.

